
JOURNAL INFORMATION
==============================
NLM Title Abbreviation: Biospektrum (Heidelb)
Journal ID: Biospektrum (Heidelb)
NLM Journal ID: 9615685

Biospektrum

ISSN: 0947-0867
EISSN: 1868-6249

ARTICLE INFORMATION
==============================
PMCID: PMC7880633
PMID: 33612989
DOI: 10.1007/s12268-021-1512-x
Article ID: 1512
Article version: 1
Subjects: Wissenschaft Special

COR-101, ein menschlicher Antikörper gegen COVID-19

Dübel Stefan biotech@tu-bs.de http://www.tu-braunschweig.de/bbt/biotech/corat-corona-antibody-team
1
Herrmann Andreas 2
Schirrmann Thomas 2 3
Frenzel André 2 3
Hust Michael 1
1 grid.6738.a 0000 0001 1090 0254 Abteilung Biotechnologie, Institut für Biochemie, Biotechnologie und Bioinformatik, Technische Universität Braunschweig, Spielmannstraße 7, D-38106 Braunschweig, Deutschland
2 Corat Therapeutics Gmbh, Braunschweig, Deutschland
3 Yumab GmbH, Braunschweig, Deutschland
Electronic publication date: 2021 Feb 12
Print publication date: 2021
Volume: 27
Issue: 1
First page: 46
Last page: 48
Copyright: © Die Autoren 2021
License: Dieser Artikel wird unter der Creative Commons Namensnennung 4.0 International Lizenz veröffentlicht, welche die Nutzung, Vervielfältigung, Bearbeitung, Verbreitung und Wiedergabe in jeglichem Medium und Format erlaubt, sofern Sie den/die ursprünglichen Autor(en) und die Quelle ordnungsgemäß nennen, einen Link zur Creative Commons Lizenz beifügen und angeben, ob Änderungen vorgenommen wurden. Die in diesem Artikel enthaltenen Bilder und sonstiges Drittmaterial unterliegen ebenfalls der genannten Creative Commons Lizenz, sofern sich aus der Abbildungslegende nichts anderes ergibt. Sofern das betreffende Material nicht unter der genannten Creative Commons Lizenz steht und die betreffende Handlung nicht nach gesetzlichen Vorschriften erlaubt ist, ist für die oben aufgeführten Weiterverwendungen des Materials die Einwilligung des jeweiligen Rechteinhabers einzuholen. Weitere Details zur Lizenz entnehmen Sie bitte der Lizenzinformation auf http://creativecommons.org/licenses/by/4.0/deed.de.
License URL: https://creativecommons.org/licenses/by/4.0/

issue-copyright-statement: © Springer-Verlag GmbH Deutschland, ein Teil von Springer Nature 2021

==============================
COR-101 is a fully human, Fc silenced IgG that was discovered by antibody phage display. It reduced the SARS-CoV-2 virus load in the lung by more than 99 percent in Hamster models and led to much faster recovery. Its mode of action has been elucidated by solving the atomic structure of its interaction with SARS-CoV-2. The antibody competes with ACE2 binding by blocking a large area of the SARS-CoV-2 spike protein.

■ Bereits vor fünf Jahren wurden wir gewarnt. „Unsere Arbeiten zeigen ein mögliches Risiko, dass SARS-CoV aus zirkulierenden Viren in Fledermaus-Populationen wieder hervorbrechen kann“, stellten Menachery *et al.* [1] fest. Vier Jahre später wurde eine neuartige schwere Lungenerkrankung (SARS-CoV-2) beschrieben, die von einem neuen Corona Virus (SARS-CoV-2) hervorgerufen wird [2]. SARS-Cov-2 ist ein RNA-Virus und bindet mittels seines Spike-Hüllproteins (S) an menschliche Zellen mit dem Angiotensin-Converting-Enzyme-2-Rezeptor (ACE2), dringt in sie ein und kann sich dort vermehren. Die Rezeptorbindungsdomäne (RBD) innerhalb des Spike-Proteins ist für die direkte Bindung an ACE2 verantwortlich [3]. Eine direkte Blockade der ACE2-RBD-Bindung kann die Infektion und somit die Vermehrung von SARS-CoV-2 verhindern. Für diese Art der Immuntherapie eignen sich besonders menschliche rekombinante Antikörper (*fully human antibodies*), da diese Molekülklasse zum einen identisch zu einem der natürlichen Abwehrmechanismen unseres Körpers gegen neue unbekannte Viren ist und zum anderen ihre pharmazeutischen Eigenschaften, insbesondere gute Verträglichkeit, gut bekannt sind. Auch ihre biotechnologische Herstellung und Logistik sind robust und seit Jahrzehnten bestens etabliert. Aktuell sind mehr als 90 Antikörper von der EMA und FDA zugelassen, die meisten für die Krebstherapie und Autoimmunkrankheiten. Antikörper gegen beliebige, auch vorher unbekannte Antigene können heute sehr schnell identifiziert werden,

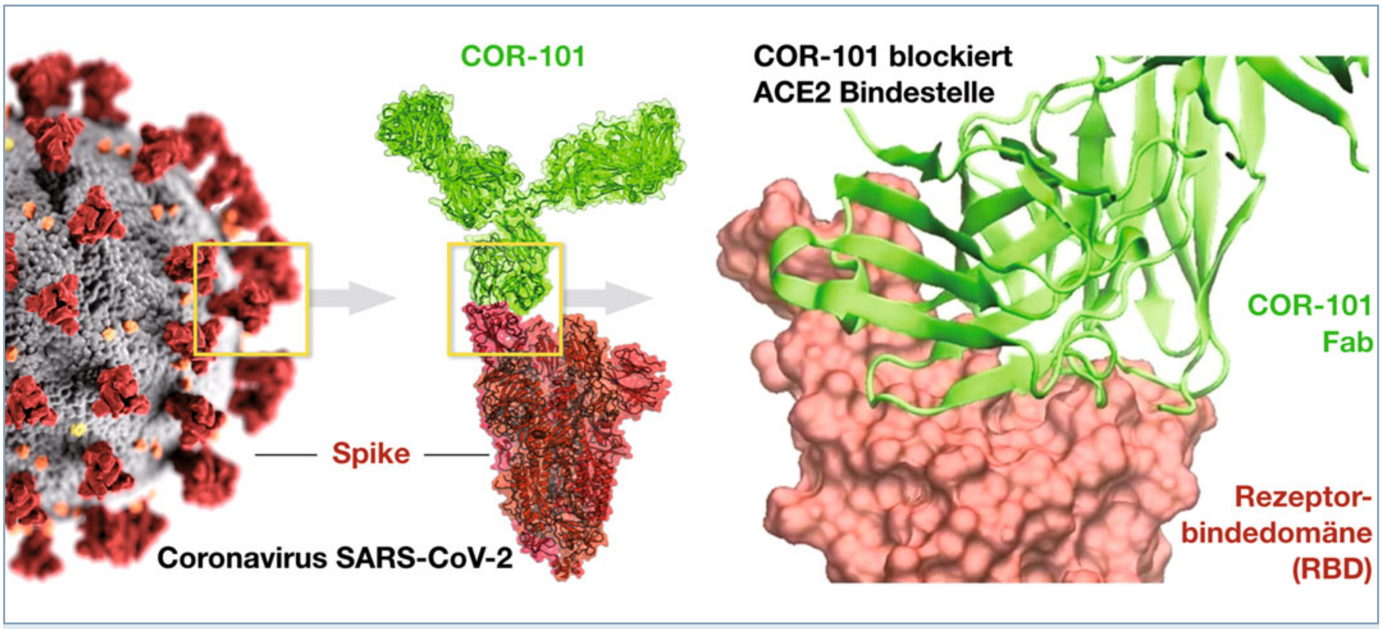

▲ **Abb. 1:** Die Kristallstrukturanalyse der Bindung von COR-101 (grün) an SARS-CoV-2 (rot) zeigt, wie der Antikörper die Bindungsstelle des Virus-Spike-Proteins an menschliche Zellen sterisch komplett überdeckt und so blockiert. PDB-Datei mit den Strukturdaten: 7B3O (www.rcsb.org).

insbesondere aus einzelnen B-Zellen oder durch *in vitro*-Technologien, wie dem Antikörper-Phagendisplay [4].

## Impfstoffe können COVID-19 nicht heilen

Ein Heilmittel zur Behandlung von COVID-19 wird auch nach dem Start der SARS-CoV-2-Impfungen weiterhin dringend benötigt. Die Impfungen können weder Erkrankte heilen noch in absehbarer Zeit alle Menschen davor schützen, weil dies eine mehrfache Immunisierung von wahrscheinlich mehr als der Hälfte der Menschen voraussetzt. Die Dauer der Wirksamkeit der Impfstoffe ist zudem noch unbekannt. Ebenso ist noch keinesfalls klar, wie gut der Impfstoff bei Älteren wirkt und bei Menschen, die aus medizinischen Gründen immunsupprimiert sind. Auch ist noch nicht vollständig gesichert, dass die Impfung die Transmission, also die Übertragung von Mensch zu Mensch, ausreichend effizient unterbindet, um die Pandemie zu stoppen. Unabhängig davon hängt viel davon ab, wieviel Personen überhaupt bereit sind, sich impfen zu lassen. Es wird deshalb in jedem Fall Jahre dauern, bis ein ausreichender Impfschutz in der gesamten Bevölkerung erreicht wird. In dieser Zeit ermöglichen Antikörperwirkstoffe nicht nur die Behandlung der Erkrankten, sondern auch den sofortigen prophylaktischen Schutz von Risikogruppen, oder eine vorbeugende Behandlung nachdem durch Kontaktverfolgung eine mögliche Ansteckung bekannt wurde.

Mittels Antikörper-Phagendisplay [4] konnten wir in der Vergangenheit bereits auch neutralisierende Antikörper gegen das Sudan-Ebola-Virus [5], das Westliche Pferde-Enzephalitis-Virus (WEEV, [6]) oder das Marburg-Virus [7] entwickeln. In der aktuellen Pandemie haben wir menschliche Antikörper gegen SARS-CoV-2 aus dem gesamten Antikörpergenrepertoire von B-Zellen von gesunden Spendern, aber auch genesenden COVID-19-Patienten mittels Antikörper-Phagendisplay gewonnen. Aus beiden Quellen wurden zahlreiche Antikörper erhalten, die die Bindung von Virus-RBD an ACE2 auf den Wirtszellen effizient blockieren und die Infektion der SARS-CoV-2-Viren verhindern [8, 9]. Einen Überblick über die Strategie gibt [10].

## COR-101 schützt vor SARS-CoV-2 durch Blockierung der Infektion

Der Antikörper COR-101 (STE90-C11, [9]) wurde nach umfangreichen Tests aufgrund seiner optimalen Aktivität und Produkteigenschaften für die klinische Entwicklung ausgewählt. Er blockiert nicht nur die Bindung des Spike-Proteins an ACE2, sondern erkennt auch zahlreiche SARS-CoV-2 Mutanten. Die Struktur dieses besonderen Antikörpers und die Bindung an die RBD wurden mittels Röntgenkristallographie aufgeklärt. COR-101 blockiert großflächig genau die Stelle in der SARS-CoV-2-RBD, wo diese an ACE2 binden würde ([9], **Abb. 1**). Diese Eigenschaft könnte sich als vorteilhaft gegenüber Antikörpern erweisen, die Ende 2020 die Notfallzulassung in den USA bekommen haben. Ein weiterer Unterschied ist, dass der konstante Fc-Teil von COR-101 derart optimiert wurde, dass er nicht mehr das Immunsystem durch Bindung an Fc-Gamma Rezeptoren oder Komplement aktivieren kann. Damit wird die Gefahr einer möglichen zusätzlichen Entzündungsreaktion, die die Lunge schädigt, stark verringert. Im Hamstermodel reduzierte die Behandlung mit COR-101 den Virustiter in der Lunge nach drei Tagen im Vergleich zur unbehandelten Kontrollgruppe um mehr als 99 Prozent. Die Tiere verloren weit weniger Gewicht und wurden deutlich schneller gesund als die der Kontrollgruppe. COR-101 kann also das Virus im lebenden Organismus effizient neutralisieren und das Fortschreiten der Krankheit verhindern. Das verbesserte Design des Fc-Teils soll auch die Gefahr einer möglichen Antikörper-vermittelten Verstärkung (*antibody dependent enhancement*, ADE) des Virus reduzieren. Aus diesem Grund wird COR-101 nicht nur zur Behandlung milder Erkrankungsverläufe, sondern auch zur Behandlung schwerer erkrankter Patienten erprobt. Hocheffiziente und schnelle Verfahren zur Herstellung von COR-101 wurden entwickelt und die Chargen nach Medikamentenstandards (GMP, *good manufacturing practice*) für die ersten klinischen Studien (NCT04674566 auf www.clinicaltrials.org) bereits im November fertiggestellt.

## Danksagung

Besonders bedanken wir uns beim Land Niedersachsen und der Deutschen Herz-

Hier steht eine Anzeige.

Springer stiftung für die finanzielle Förderung. Diese Arbeit wäre ohne den außergewöhnlichen Einsatz der wissenschaftlichen und technischen Mitarbeiter der beteiligten Arbeitsgruppen und die fantastische Unterstützung zahlreicher Forscher, Ärzte und Patienten aus ganz Deutschland nicht möglich gewesen. Ihnen allen ist dieser Artikel gewidmet.

ARBEITSGRUPPE

CORAT (*Co*rona *A*ntibody *T*eam) wurde von Stefan Dübel (TU Braunschweig) und seinem Kollegen Michael Hust zusammen mit Gundram Jung (Univ. Tübingen) und der YUMAB GmbH initiiert. Das Konsortium von mehr als 50 Wissenschaftlern und Ärzten aus akademischen Institutionen und der Industrie hat gemeinsam das COVID-19-Medikament COR-101 entwickelt, welches die CORAT Therapeutics GmbH nun klinisch testet. Das Bild zeigt, stellvertretend für das ganze Corona Antibody Team und die CORAT Therapeutics GmbH, die TAs, Doktoranden und Postdocs der TU Braunschweig, welche die extrem schnelle Entwicklung von COR-101 mit ermöglicht haben.

Hier steht eine Anzeige.

Springer


Funding note

Open Access funding enabled and organized by Projekt DEAL.


Literatur

[1] Menachery VD Yount BL Debbink K A SARS-like cluster of circulating bat coronaviruses shows potential for human emergence Nat Med 2015 21 1508 1513 10.1038/nm.3985 26552008 PMC4797993
[2] Zhou P Yang XL Wang XG A pneumonia outbreak associated with a new coronavirus of probable bat origin Nature 2020 579 270 273 10.1038/s41586-020-2012-7 32015507 PMC7095418
[3] Wang Q Zhang Y Wu L Structural and functional basis of SARS-CoV-2 entry by using human ACE2 Cell 2020 181 894 904 10.1016/j.cell.2020.03.045 32275855 PMC7144619
[4] Frenzel A Kügler J Helmsing S Designing human antibodies by phage display Transfus Med Hemother 2017 44 312 318 10.1159/000479633 29070976 PMC5649246
[5] Froude JW Herbert AS Pelat T Postexposure protection in mice against sudan virus by a two antibody cocktail Viruses 2018 10 286 10.3390/v10060286 PMC6024315 29861435
[6] Burke CW Froude JW Miethe S Human-like neutralizing antibodies protect mice from aerosol exposure with western equine encephalitis virus Viruses 2018 10 147 10.3390/v10040147 PMC5923441 29587363
[7] Froude JW Pelat T Miethe S Generation and characterization of protective antibodies to Marburg virus mAbs 2017 9 696 703 10.1080/19420862.2017.1299848 28287337 PMC5419075
[8] Bertoglio F, Meier D, Langreder N et al. (2020) SARS-CoV-2 neutralizing human recombinant antibodies selected from pre-pandemic healthy donors binding at RBD-ACE2 interface. Nat Commun, im Druck10.1038/s41467-021-21609-2 PMC7952403 33707427
[9] Bertoglio F, Fühner V, Ruschig M et al. (2020) A SARS-CoV-2 neutralizing antibody selected from COVID-19 patients by phage display is binding to the ACE2-RBD interface and is tolerant to known RBD mutations. Nat Commun, im Druck, https://papers.ssrn.com/sol3/papers.cfm?abstract_id=3754550 10.1016/j.celrep.2021.109433 PMC8260561 34273271
[10] Dübel S Hust M Frenzel A Rekombinante, vollständig humane Antikörper zur Behandlung akuter COVID-19 BIOspektrum 2020 4 44 446 10.1007/s12268-020-1404-4 PMC7318721 32834543
